# Improving the quality of needle and syringe programmes: an overlooked strategy for preventing hepatitis C among people who inject drugs

**DOI:** 10.1186/1471-2334-14-S6-S8

**Published:** 2014-09-19

**Authors:** Astrid Leicht

**Affiliations:** 1 Fixpunkt e. V., Berlin, Germany

## Introduction

The hepatitis C virus (HCV) is 5 to 20 times more infectious than HIV [1]. Even more alarmingly, HCV has the capacity to survive outside of the human body for weeks. Unfortunately, this makes the reuse of injecting equipment that has been contaminated with HCV a highly effective means of spreading the disease [2]. The prevalence of HCV among people who inject drugs (PWID) is shocking, much higher than HIV prevalence, for example [3,4].

Research suggests that needle and syringe programmes (NSPs) contribute less to the prevention of HCV than the prevention of HIV [5]. Nevertheless, the distribution of needles and syringes has become an accepted worldwide strategy to prevent the spread of not only HIV but also other bloodborne infectious diseases such as HCV among people who inject drugs.

NSPs and opioid substitution therapy are key recommended interventions of multilateral organisations such as the World Health Organisation (WHO), the Joint United Nations Programme on HIV/AIDS, the United Nations Office on Drugs and Crime, the European Centre for Disease Prevention and Control, and the European Monitoring Centre for Drugs and Drug Addiction (EMCDDA) [3,6,7]. Yet NSPs are relegated to the sidelines in most European countries. They are neglected by government officials and funders, and are even often underprioritised by drug treatment organizations.

Despite the valuable efforts of EMCDDA, key European stakeholders have shown little interest in formulating uniform data collection procedures, minimum quality standards and best practices relating to disease prevention interventions for drug users. This means that experiences in the field are not sufficiently evaluated, and information is lacking on how to further develop programmes to demonstrate and increase effectiveness. Scotland is one of the few favourable exceptions in Europe [8].

Embarrassingly little information exists about the effectiveness of NSPs – an evidence gap that stands out all the more in comparison to the body of research on medical-therapeutic interventions for hepatitis C infection. Given the limited interest in NSPs among scientific experts and practitioners working in the field of harm reduction, many outstanding questions and challenges remain in regard to optimal procedures for using NSPs as an HCV prevention tool.

## Needle and syringe distribution: good practices

In 2013, the Australian National Council on Drugs published a position paper identifying a number of factors that can improve the performance of NSPs [9]. The long-term experience of Fixpunkt, a nongovernmental organisation in Germany, confirms the Council’s findings regarding the importance of the following:

• NSP programmes need to be well accepted by government officials, funders and the police as an effective strategy to prevent disease transmission, and also by the staff who manage the implementing organizations.

• A range of NSP service modalities should be utilised including primary NSPs, peer-based (secondary) NSPs, pharmacy-based services, syringe vending machines, mobile and outreach services, and distribution in prison settings.

• Supplies and equipment should be made available based upon the needs of consumers (non-injecting and injecting drug users).

• Staff of NSPs must be well informed, well trained, and prepared to take into account service issues relating to stigma, gender and cultural diversity.

• Programmes should offer expert counselling on how to use (or not use) the prevention equipment and materials provided based on individualised risk assessments and motivation.

• Additional prevention services need to be offered, including HIV/HCV rapid testing, vaccination against hepatitis A and hepatitis B, consumption control/reduction programs, overdose prevention, and interventions to promote transition to non-invasive forms of drug application (inhaling, snorting, rectal application).

• Counselling and referrals to opioid substitution treatment, addiction-related therapies and medical treatment should be provided, with an emphasis on treating hepatitis C.

## Next steps

It is essential to raise awareness and interest among service providers and scientific researchers in order to improve data collection, evaluation, reflection and dissemination of quality standards for effective needle/syringe programmes. WHO recommendations are the basis for this [10,11]. They must be adapted and formalised as guidelines (not only “recommendations”) in accordance with national and local conditions and needs.

New and changing injection behaviours must be taken into account when designing and developing NSP services and interventions. Many of these trends warrant further research and exploration. Examples include the transition from heroin to methamphetamine use which recently occurred in Israel and Romania [11-14], the injection of performance-enhancing drugs (steroids), and the injection of amphetamines and methamphetamines among men who have sex with men in the context of sexual risk-taking behaviour [15,16].

Last but not least, researchers, health officials and NSP service providers must be open-minded and explore the wider context of motivations among drug injectors in order to prevent infections and promote health and well-being more broadly in this population.

## Competing interests

The authors declare that they have no competing interests.

## Declarations

This article has been published as part of *BMC Infectious Diseases* Volume 14 Supplement 6, 2014: Viral Hepatitis in Europe. The full contents of the supplement are available online at http://www.biomedcentral.com/bmcinfectdis/supplements/14/S6. The publication charges for this supplement were funded by AbbVie as an unrestricted grant to Rigshospitalet, the University of Copenhagen. AbbVie further funded the printing of the supplement with additional financial support from the Drug Prevention and Information Programme (DPIP) of the European Union.
